# ‘E-learning’ modalities in the current era of Medical Education in Pakistan

**DOI:** 10.12669/pjms.305.4351

**Published:** 2014

**Authors:** Masood Jawaid, Syed Moyn Aly

**Affiliations:** 1 Dr. Masood Jawaid, MCPS, MRCS, FCPS, Assistant Professor Surgery and Incharge e-Learning, Dow University of Health Sciences, Karachi – Pakistan; 2 Dr. Syed Moyn Aly, MBBS, MHPE, Chair, Department of Medical Education, College of Medicine, Taif University, Taif, Saudi Arabia

**Keywords:** e-learning, Medical education, e-learning modalities

## Abstract

There are a number of e-Learning modalities, some or all of which may be used throughout a medical, dental, nursing or any other health related undergraduate curriculum. The purpose of this paper is to briefly describe what e-learning is along with some of the modalities, their common advantages and limitations. This publication ends with practical implications of these modalities for Pakistan.

From the stark mountainous terrains of Balochistan to the lush valleys of Kashmir, wherever there are medical colleges, public or private, there are students using smart phones, iPads and other electronic devices. The use of such e-devices, in Pakistan, seems mostly for entertainment purposes. The use of such gadgets is yet to percolate the academic strata and is yet to become common across the length and breadth of our country. The purpose of this paper is to briefly discuss what e-learning is and describe some of the modalities along with common advantages and limitations and their practical implication for Pakistan.

In 2001, Marc Rosenberg suggested the following definition of eLearning: “The use of internet technologies to deliver a broad array of solutions that enhance knowledge and performance”.^1^ However e-learning is more than the use of internet technologies. In 2003 another definition was proposed by Derek Stockley. “E-learning involves the use of a computer or electronic device (e.g. a mobile phone) in some way to provide training, educational or learning material”.^2^ One of the recent definitions of eLearning is: “Learning in which some content or activity is delivered via computers *in any way*, sometimes to the learning of content from the internet and sometimes to using a virtual learning environment (VLE)”.^3^

There are a number of e-Learning modalities, some or all of which may be used throughout a medical, dental, nursing or any other health related undergraduate curriculum.

**Asynchronous audio or video:** ^4^ Teachers can post audio (podcast as mp3 files) or video files (vodcast) These files are automatically sent to a subscriber’s computer or mobile hand held device like smart phones. There needs to be a centrally placed distributor called podcatcher which is responsible for sending the files to the subscribers (students).

**Blended Learning:** ^5^ Online or computer based learning with face to face teaching is called blended learning. The goal of blended learning is to provide the most efficient and effective instruction experience by combining different delivery modalities.

**Chat / Video Conference:** ^3^ Chat can be in the form of text-only, audio or audio and video (e.g. Skype). Chats can include or be supervised by teacher or it can be among students only (for example, group discussions for online collaboration). 

**Computer-aided Learning (Courseware):** ^6^ These are either online or most commonly media based textual, pictorial, video or interactive exercises with self-assessment questions and immediate feedback.

**Computer Based Tests:** ^7^ Instead of having scenarios (A-type), MCQs can have audio, video or flash animations to improve their validity. Tests can be given with time limit and attempt restrictions. It’s a very important tool especially for formative individualized feedback to students.

**Educational online games / experiences:** ^8^ There is a large collection of online educational games; they allow students to interact and receive feedback from the game/ activities.

**ePBL:** ^9^ A case is created and distributed through email or VLE. Students interact with each other and with a facilitator via chat room, forum, email or whiteboard. The facilitator may take the role of the traditional facilitator or role-play the characters in the case.

**ePortfolio:** ^10^ The learner builds and maintains a digital repository of his activities and achievements online, which they can use to demonstrate competence and reflects on their learning.

**Online collaboration (Wikis, Blogs and Interactive Whiteboards):** ^11^ Students work collaboratively without restriction of time and space. There are many tools to enable group collaboration online, including wikis, blogs, Google Drive, interactive whiteboards. Teachers facilitate and answer questions but usually don’t actively participate in collaborative assignments.

**Online Discussions Forums:** ^12^ Discussions can be started on a specific topic by a student or faculty member and others can reply to the issue posted (threaded discussions). There are various options for how to structure these; for example conversation topics can either be assigned or open. Another type of discussion forum is Question and Answer in which teacher asked a question and student have to post first their answer before they can see other students response. It is possible to keep participation restricted for students or students and teachers. In many postgraduate courses, discussion forums are used as collaborative learning activities.

**Repository and Hypertext:** ^13^ Teachers can post readings or links to readings on a public or secure website. Readings can be in the form of html pages, PDFs, Word documents or PowerPoint etc. Hypertext can be in the form of online books, webpages of organized and interrelated materials, to random collection of text studded with links to pages all over the world.

**Synchronous audio or video:** ^14^ This could be an online broadcasting of a traditional lecture or seminar, but can also be a collective viewing of a presentation (SlideShare) with chat. Different paid and free solutions are available easily.

**Virtual classrooms:** ^15^ A virtual reality space (Second Life) can be used to resemble a traditional real life classroom. Students and teachers create avatars (online representations of their own) and login online simultaneously in the virtual space. The teacher can then lead a usual lecture or small group discussion or an entirely new, multimedia event can be created for more learning engagement.

**Virtual learning Environments (VLE):** ^16^ These are frameworks into which learning material with different activities are inbuilt. In VLE teaching and administrative tools are available in a single system. The activities present in a typical VLE are forums, chats, lessons, wikis, blogs, assignments and quizzes etc. All VLE can incorporate third party modules with SCORM packages. VLE are either open source (Moodle, Sakai) or commercial (Blackboard, WebCT).

**Pros and cons of e-Learning for institutions of Pakistan: **An institute needs to have a clear understanding of the e-learning modalities available and then select the one that suits its needs and matches its resources; what suits one institute or what is common may not always fit one’s another’s needs! It needs to be sure why it wants an e-learning modality, what benefits it will get and what it will have to invest (in terms of financial and human resources) in order to make the venture useful educationally.

 Most of the modalities mentioned above are finance-intensive; they require heavy investment, but the long term results would be worthwhile. The institute (wishing to use an e-learning modality, e.g. LMS) will need to recruit qualified and preferably experienced faculty members and related computer systems. 

 Such personnel will have to train faculty and students in how to use the system; faculty and student orientation and development are a must for all stakeholders to understand and use the system efficiently and effectively. This faculty development process must be on-going and not a once-a-year event. 

 It is highly advisable to start with the simplest (e.g. webinars or online lectures) so that the faculty and the students get accustomed to using innovative computer-based technology for routine academic and administrative work.

## SUMMARY

In a country, now increasingly relying on self-generated electricity, employing computer-based learning systems needs careful consideration for judicious and maximal use. To stay in-line with educational development within and outside the country, health education related institutes need to understand the benefits and limitations of such learning systems. Starting from simple and moving on to more sophisticated systems (over a matter of years), will boost the process of learning and student motivation, equip the faculty with enhanced skills in the use of computer-based education and assist the administration in efficient educational management. 

A key issue, which needs to be researched, is how many institutions in Pakistan are using eLearning modalities officially, why they chose the system that they did and to what benefit. It also seems to be important to find out *why *some institutions are not using them.Developing a database of such information would help institutions collaborate and merge resources for the greater interest of medical education and ultimately for health care delivery.

## Notes

***Source of Funding:*** Nil.
